# Analysis of cultivable microbiota and diet intake pattern of the long-lived naked mole-rat

**DOI:** 10.1186/s13099-016-0107-3

**Published:** 2016-05-28

**Authors:** Tewodros Debebe, Susanne Holtze, Michaela Morhart, Thomas Bernd Hildebrandt, Steffen Rodewald, Klaus Huse, Matthias Platzer, Dereje Wyohannes, Salomon Yirga, Alemayehu Lemma, Rene Thieme, Brigitte König, Gerd Birkenmeier

**Affiliations:** Medical Faculty, Institute of Biochemistry, University of Leipzig, Johannisallee 30, 04103 Leipzig, Germany; Medical Faculty, Institute of Medical Microbiology, University of Leipzig, Leipzig, Germany; Institute of Pharmacy, University of Leipzig, Leipzig, Germany; Department of Visceral, Transplantation, Thoracic and Vascular Surgery, University Medical Center Leipzig, University of Leipzig, Leipzig, Germany; Department of Reproduction Management, Leibniz-Institute for Zoo and Wildlife Research, Berlin, Germany; Leibniz Institute on Aging-Fritz Lipmann Institute, Jena, Germany; College of Natural Sciences, Addis Ababa University, Addis Ababa, Ethiopia; College of Veterinary Medicine and Agriculture, Addis Ababa University, Addis Ababa, Ethiopia; College of Medicine and Health Sciences, Bahir Dar University, Bahir Dar, Ethiopia

**Keywords:** Naked mole-rat, Microbiota, Diet, Polyphenols

## Abstract

**Background:**

A variety of microbial communities exist throughout the human and animal body. Genetics, environmental factors and long-term dietary habit contribute to shaping the composition of the gut microbiota. For this reason the study of the gut microbiota of a mammal exhibiting an extraordinary life span is of great importance. The naked mole-rat (*Heterocephalus glaber*) is a eusocial mammal known for its longevity and cancer resistance.

**Methods:**

Here we analyzed its gut microbiota by cultivating the bacteria under aerobic and anaerobic conditions and identifying their species by mass spectrometry.

**Results:**

Altogether, 29 species of microbes were identified, predominantly belonging to Firmicutes, and Bacteroidetes. The most frequent species were *Bacillus megaterium* (45.2 %), followed by *Bacteroides thetaiotaomicron* (19.4 %), *Bacteroides ovatus*, *Staphylococcus sciuri* and *Paenibacillus* spp., each with a frequency of 16.1 %.

**Conclusion:**

Overall, the gut of the naked mole-rat is colonized by diverse, but low numbers of cultivable microbes compared with humans and mice. The primary food plants of the rodents are rich in polyphenols and related compounds, possessing anti-microbial, anti-inflammatory, anti-oxidative as well as anti-cancer activity which may contribute to their exceptionally healthy life.

**Electronic supplementary material:**

The online version of this article (doi:10.1186/s13099-016-0107-3) contains supplementary material, which is available to authorized users.

## Background

The microbiota is defined as the entity of microbial communities, which colonize different parts of the body of a host organism. These habitats include among others, the gut, oral cavity, skin, eyes and vagina [1]. Increasing evidence supports the notion that the microbiota has a strong influence on health and disease in humans [2, 3]. Recent studies have concluded that the composition and function of microbiota play a significant role in autoimmunity and immune regulation, development of cancer, Crohn’s disease, obesity, and type 1 and 2 diabetes [4–7]. This causal relationship came currently in the focus of scientific interest. Analyzing the composition, function, and distribution of the microbiota in relation to diet, environment, genetic factors, and host immunity will help to elucidate the pivotal role of the microbiota in health and pathogenesis of diseases. The naked mole-rat (*Heterocephalus glaber*) is a eusocial mammalian species that lives in colonies of up to 300 individuals. For this mouse-sized subterranean rodent a life span of about 30 years has been reported for both, reproductive and non-reproductive castes. Moreover, it is known to appear healthy over its life span, displays resistance to oxidative stress and is remarkably resistant to both spontaneous cancer and experimentally induced tumorigenesis [8]. However, the recent report of two cases of cancer in zoo-housed naked mole-rats may not alter the longstanding observation of cancer resistance of these animals [9]. A recent study has shown that captive naked mole-rats have higher levels of oxidative damage and decreased level of anti-oxidants compared to short-lived mice, indicating the involvement of further mechanisms known to counter high levels of oxidative damage [10]. Thus, naked mole-rats pose a challenge to the current theories that link aging, cancer and redox homeostasis. For these reasons as well as their close phylogenetic relation to humans, naked mole-rats are of special interest in the search for mechanisms leading to a particularly long and healthy life [11]. Recent publications demonstrate a strong link between different diseases and gut microbiota. This prompted us to investigate the gut microbiota of the naked mole-rat to obtain deeper insights into their astonishing longevity. Therefore, for the first time, we analyzed and characterized cultivable gut microbes obtained from the intestine and feces of wild naked mole-rats. Furthermore, we assessed the main diet of the animal in the wild and discuss their constituents in relation to their medical significance.

## Methods

### Naked mole-rat sampling

Eleven wild naked mole-rats from the Rift Valley of Ethiopia were captured and detained. Intestinal and fecal samples of the animals were obtained from individuals captured in Ethiopia. Permission comprising both, field permit and ethics approval was granted by the Ethiopian Wildlife Conservation Authority (EWCA; ref. No. 31/394/07 dated 27 November 2014). Intestinal content and feces were collected and immediately frozen in liquid nitrogen until further analysis. Animal collection and sampling were performed in accordance with the approved guideline and regulation of the national wildlife authority of Ethiopia.

### Microbial growth conditions

Following standard aseptic procedures, samples of the colon, cecum and feces from naked mole-rats were subjected to microbial identification. One gram of intestine and feces of each animal were weighed and thoroughly dispersed and homogenized by vortex in 3 mL of sterile saline (0.85 %) (bioMérieux, Marcy I’Etoile, France) for 1 min and tenfold serial dilutions were made subsequently. Next, from each dilution 0.1 mL aliquot was plated onto blood agar (Carl Roth GmbH, Karlsruhe, Germany) and Brucella blood agar (Becton, Dickinson, Sparks, MD, USA) and incubated at 37 °C for 24–72 h at ambient air or anaerobically, respectively. Following incubation, the growth of bacteria was recorded and the number of colonies was counted based on their morphology. The colonies were further sub-cultured individually to obtain pure cultures. Subsequently, each of the pure colonies was identified by matrix-assisted laser desorption ionization time-of-flight (MALDI-TOF) mass spectrometry and gram staining was performed for each pure culture as a cross check with the MALDI-TOF results.

### MALDI-TOF-MS based microbe identification

The automated MALDI-TOF was performed following the standard protocol (bioMérieux, Marcy I’Etoile, France). Freshly grown pure microbial cells from a single colony and a control (*Escherichia coli*) were deposited onto the target slides and to each, 1 µL of the α-cyano-4-hydroxycinnamic acid (CHCA) matrix solution (VITEK^®^ MS-CHCA REF 411071, bioMérieux, Marcy I’Etoile, France) was added. After solvent evaporation at room temperature, the slides were inserted into the device and analyzed (VITEK^®^-MS, Marcy I’Etoile, France). Finally, sample spectra were compared to an extensive database of known bacterial species by VITEK^®^-MS proofing, allowing us to accurately identify the microorganism in question.

### Microbial inoculum preparation

*Bacillus megaterium*, *Staphylococcus scuiri*, *Parabacteroides distasonis*, *Clostridium ramosum*, *Bacteroides ovatus* from the naked mole-rat and two clinical human pathobionts, *E. coli* and *Staphylococcus aureus* were maintained on blood agar or Brucella blood agar at 37 °C for 24–48 h aerobically or anaerobically, respectively. Following incubation, isolates were adjusted to McFarland 0.5 (~10^8^ cells/mL) in a 0.85 % saline solution. Subsequently, standardized microbial suspension at low (10^3^ cells/mL) and high (10^6^ cells/mL) concentrations were prepared in Roswell Park Memorial Institute (RPMI)-1640 medium.

### Whole blood assay

Heparinized blood was obtained from healthy human volunteers and stimulated by lipopolysaccharide (LPS) *E. coli* 0111:B4 (10 ng/ml) (SigmaAldrich; Taufkirchen, Germany) as previously described [12]. Standardized live microbial suspension at low (10^3^ cells/mL) or high (10^6^ cells/mL) final concentrations were mixed with 200 µL heparinized human blood contained in a total volume of 800 µL RPMI-medium in 24-well culture plates (Cellstar^®^ Greiner Bio-One, Frickenhausen, Germany) at 37 °C and 5 % CO_2_ for 8 h. Following incubation, the suspension was transferred into fresh sterile 1.5 mL micro centrifuge tubes and centrifuged at 13,000×*g* for 5 min. Finally, plasma supernatants were harvested and stored at −20 °C. The local ethics committee of the Faculty of Medicine of the University of Leipzig, Germany, approved this study in accordance to the ICH-GCP guidelines (Reference No.: 057-2010-08032010). Written informed consent was obtained from all subjects and the experiments were performed in accordance with the guidelines and regulation of the Medical Faculty of the University of Leipzig.

### Cytometry bead array assay

Assessment of cytokine levels in plasma supernatants of human blood cell cultures was performed according to the manufacturer’s instruction using cytometry bead array (CBA) immunoassay as previously described [12]. The data were analyzed using a FACS Calibur and the CellQuest™ Software (BD Biosciences).

### Feeding trials and analysis of biochemical constituents of the wild naked mole-rat’s diet

Different plant species that are distributed nearby the habitat of the animals were freshly collected. Feeding trials were performed on captured members of the colony temporarily managed near Hawassa, South Ethiopia. Within this trial the degree of consumption of the plant species offered to the animals was recorded and categorized as either main diet, or rarely consumed food source. In order to investigate the constituents and medical significance of the plant species consumed by the animals in the wild, a systematic review of the appropriate literature was performed. At first, the scientific name of all plant species was verified. To fulfill this special assignment, we used the dynamic checklist of the catalogue of life (www.catalogueoflife.org). For analyzing the chemical content of identified plants the database scifinder (www.cas.org) was used. Dependent on the subject of search, the number of the proven sources differed from nil to over 10,000, for instance the common crops *Ipomoea batatas* (sweet potato) and *Arachis hypogaea* (groundnut) are well-described whereas two plants, *Aloe trichosantha* and *Endostemon tenuiflorus* are rarely described. Therefore, for these species the chemical content and bioactive substances were suggested by comparing them with the higher-ranking genus and family only [13, 14].

### Data analysis

The data obtained were analyzed in terms of descriptive statistics. Characteristics of each isolates, the constituent and medicinal values of plant species were systematically reviewed. One way ANOVA and Student’s *t* test were used to analyze the mean difference in cytokine levels for each group of microbes compared with negative control and LPS-stimulated samples using GraphPad Prism version 5 (San Diego, CA, USA) and plotted as mean ± SEM. p value <0.05 was considered statistically significant.

## Results

### Frequency of microbes isolated from wild naked mole-rats

In the present study, we identified the composition of gut microbes of the naked mole-rat (Table 1). Overall, 29 different species of microbes were identified from the colon, cecum and feces of wild naked mole-rats. The principal microorganisms of the naked mole-rats belong to the phylum Firmicutes (58.6 %), followed by Bacteroidetes (20.7 %). Less frequently identified phyla were, Proteobacteria (10.3 %), Actinobacteria (6.9 %) and Ascomycota (3.5 %). The amounts of cultivable microbes ranged from 10^2^ to 5 × 10^5^, 10^2^–10^5^, and 10^2^–3 × 10^5^ c.f.u./g in cecum, colon, and feces, respectively. In total, the most frequently occurring species were *B. megaterium* (45.2 %), followed in order by *Bacteroides thetaiotaomicron* (19.4 %), *B. ovatus*, *Paenibacillus* spp., *Staphylococcus scuiri* each with a frequency of 16.1 %, *Staphylococcus gallinarum* (12.9 %) and *Enterobacter cloacae*/*asburiae* (12.9 %). The remaining isolates were least identified with percentages ranging from 3.2 to 9.7 %.

**Table 1:** Distribution of the identified microbiota from the wild naked mole-rats (n = 11)

Sites	Species	Phylum	(c.f.u./g) in range	Characterstics^a^
Colon	*Staphylococcus sciuri*	Firmicutes	1 × 10^2^–10^3^	Normal flora
	*Staphylococcus cohnii* spp. *urealyticus*	Firmicutes	1–2 × 10^3^	Normal flora
	*Staphylococcus arlettae*	Firmicutes	3 × 10^2^	Normal flora
	*Staphylococcus gallinarum*	Firmicutes	2 × 10^2^–3 × 10^3^	Normal flora
	*Staphylococcus hyicus*	Firmicutes	3 × 10^2^	Normal flora
	*Staphylococcus xylosus*	Firmicutes	3 × 10^2^	Normal flora
	*Bacillus megaterium*	Firmicutes	0.2–1 × 10^4^	Normal flora
	*Bacillus subtilus*/*amyloliquefaciens*	Firmicutes	1 × 10^2^	Normal flora
	*Bacillus pumilus*	Firmicutes	1 × 10^3^	Normal flora
	*Bacillus simplex*	Firmicutes	2 × 10^3^	Normal flora
	*Paenibacillus* spp.	Firmicutes	2–3 × 10^2^	Normal flora
	*Paenibacillus pabuli*	Firmicutes	1 × 10^2^	Normal flora
	*Bacteroides ovatus*	Bacteroidetes	1–2 × 10^3^	Normal flora
	*Bacteroides thetaiotaomicron*	Bacteroidetes	1–4 × 10^5^	Normal flora
	*Parabacteroides distasonis*	Bacteroidetes	1 × 10^3^	Normal flora
	*Bacteroides vulgatus*	Bacteroidetes	5 × 10^5^	Normal flora
	*Kluyvera ascorbata*	Proteobacteria	3 × 10^3^	Normal flora
	*Klebsiella pneumoniae*	Proteobacteria	2 × 10^2^	Normal flora
	*Enterobacter cloacae*/*asburiae*	Proteobacteria	5 × 10^3^–10^5^	Normal flora
	*Actinomyces viscosus*	Actinobacteria	1 × 10^3^	Normal flora
Cecum	*Bacillus megaterium*	Firmicutes	2–4 × 10^3^	Normal flora
	*Staphylococcus gallinarum*	Firmicutes	0.4–1 × 10^3^	Normal flora
	*Staphylococcus xylosus*	Firmicutes	3 × 10^2^	Normal flora
	*Staphylococcus sciuri*	Firmicutes	1 × 10^3^	Normal flora
	*Paenibacillus* spp.	Firmicutes	0.1–1 × 10^4^	Normal flora
	*Staphylococcus warneri*	Firmicutes	1 × 10^3^	Normal flora
	*Brevibacillus* spp.	Firmicutes	4 × 10^2^	Normal flora
	*Lysinibacillus fusiformis*	Firmicutes	2 × 10^2^	Normal flora
	*Streptococcus mitis*/*oralis*	Firmicutes	2 × 10^2^	Normal flora
	*Enterococcus casseliflavus*	Firmicutes	1 × 10^3^	Normal flora
	*Bacteroides ovatus*	Bacteroidetes	0.1–1.2 × 10^4^	Normal flora
	*Bacteroides thetaiotaomicron*	Bacteroidetes	0.2–1 × 10^5^	Normal flora
	*Bacteroides fragilis*	Bacteroidetes	2 × 10^3^	Normal flora
	*Bacteroides vulgatus*	Bacteroidetes	1 × 10^5^	Normal flora
	*Enterobacter cloacae*/*asburiae*	Proteobacteria	0.7–2 × 10^3^	Normal flora
	*Actinomyces viscosus*	Actinobacteria	1 × 10^2^	Normal flora
	*Rhodococcus rhodochrous*	Actinobacteria	1 × 10^2^	Normal flora
	*Candida tropicalis*	Ascomycota	1 × 10^3^	Normal flora
Feces	*Lysinibacillus fusiformis*	Firmicutes	1 × 10^2^	Normal flora
	*Bacillus simplex*	Firmicutes	1 × 10^3^	Normal flora
	*Bacillus megaterium*	Firmicutes	0.2–3 × 10^5^	Normal flora
	*Clostridium ramosum*	Firmicutes	2 × 10^2^	Normal flora
	*Staphylococcus sciuri*	Firmicutes	1–2 × 10^3^	Normal flora
	*Staphylococcus cohnii* spp. *urealyticus*	Firmicutes	1 × 10^3^	Normal flora
	*Staphylococcus arlettae*	Firmicutes	1 × 10^2^	Normal flora
	*Bacteriodes unifromis*	Bacteroidetes	3 × 10^2^	Normal flora
	*Bacteroides thetaiotaomicron*	Bacteroidetes	2 × 10^3^	Normal flora
	*Bacteriodes ovatus*	Bacteroidetes	1 × 10^3^	Normal flora
	*Parabacteroides distasonis*	Bacteroidetes	1–3 × 10^3^	Normal flora

^a^ *Sources*: [15–19]

The intestinal content particularly colon, cecum and feces of the naked mole-rat obtained from the wild were cultivated under aerobic and anaerobic conditions. The table illustrates the species name, phylum name, cultivable amount of microbial cells and characteristics of the isolated microbiota referring to their host. Normal flora is described as a relatively stable microbial community that routinely inhabits in and on the body of a wide range of animal species. Colony-forming unit (c.f.u.).

### Occurrence of microbes within different intestinal compartments of wild naked mole-rats

The most frequently isolated species in all intestinal specimens were *B. megaterium* followed in descending prevalence by *Bacteroides thetaiotaomicron*, *B. ovatus*, and *Staphylococcus sciuri in* colon, cecum and feces. *Paenibacillus* spp., *Enterobacter cloacae*/*asburiae*, *S. gallinarum*, *Actinomyces viscosus*, *Bacteroides vulgatus*, *Staphylococcus xylosus were* identified from colon and cecum. Whereas *P. distasonis*, *Bacillus simplex*, *Staphylococcus cohnii* spp. *urealyticus*, and *Staphylococcus arlettae* were isolated from colon and feces. Except *Lysinibacillus fusiformis*, which was identified from cecum and feces, the remaining species were mono-isolates with a frequency of 3.2 % (Table 2).

**Table 2:** Frequency of microbes in colon, cecum and feces of the naked mole-rats

Species	Number of positive isolates (% isolation frequency)			
	Colon (n = 10)	Cecum (n = 11)	Feces (n = 10)	Total
*Bacillus megaterium*	5 (50)	5 (45.5)	4 (40)	14 (45.2)
*Staphylococcus sciuri*	2 (20)	1 (9.1)	2 (20)	5 (16.1)
*Bacteroides thetaiotaomicron*	2 (20)	3 (27.3)	1 (10)	6 (19.4)
*Bacillus simplex*	1 (10)	0 (0)	1 (10)	2 (6.5)
*Staphylococcus gallinarum*	2 (20)	2 (18.2)	0 (0)	4 (12.9)
*Lysinibacillus fusiformis*	0 (0)	1 (9.1)	1 (10)	2 (6.5)
*Paenibacillus* spp.	3 (30)	2 (18.2)	0 (0)	5 (16.1)
*Staphylococcus cohnii* spp. *urealyticus*	2 (20)	0 (0)	1 (10)	3 (9.7)
*Enterobacter cloacae*/*asburiae*	2 (20)	2 (18.2)	0 (0)	4 (12.9)
*Bacteroides ovatus*	1 (10)	2 (18.2)	2 (20)	5 (16.1)
*Actinomyces viscosus*	1 (10)	1 (9.1)	0 (0)	2 (6.5)
*Parabacteroides distasonis*	1 (10)	0 (0)	2 (20)	3 (9.7)
*Bacteroides vulgatus*	1 (10)	1 (9.1)	0 (0)	2 (6.5)
*Staphylococcus xylosus*	1 (10)	1 (9.1)	0 (0)	2 (6.5)
*Clostridium ramosum*	0 (0)	0 (0)	1 (10)	1 (3.2)
*Bacteroides fragilis*	0 (0)	1 (9.1)	0 (0)	1 (3.2)
*Staphylococcus arlettae*	1 (10)	0 (0)	1 (10)	2 (6.5)
*Brevibacillus* spp.	0 (0)	1 (9.1)	0 (0)	1 (3.2)
*Staphylococcus hyicus*	1 (10)	0 (0)	0 (0)	1 (3.2)
*Rhodococcus rhodochrous*	0 (0)	1 (9.1)	0 (0)	1 (3.2)
*Bacteroides unifromis*	0 (0)	0 (0)	1 (10)	1 (3.2)
*Staphylococcus warneri*	0 (0)	1 (9.1)	0 (0)	1 (3.2)
*Enterococcus casseliflavus*	0 (0)	1 (9.1)	0 (0)	1 (3.2)
*Bacillus subtilus*/*amyloliquefaciens*	1 (10)	0 (0)	0 (0)	1 (3.2)
*Bacillus pumilus*	1 (10)	0 (0)	0 (0)	1 (3.2)
*Klebsiella pneumoniae*	1 (10)	0 (0)	0 (0)	1 (3.2)
*Kluyvera ascorbata*	1 (10)	0 (0)	0 (0)	1 (3.2)
*Streptococcus mitis*/*oralis*	0 (0)	1 (9.1)	0 (0)	1 (3.2)
*Candida tropicalis*	0 (0)	1 (9.1)	0 (0)	1 (3.2)

### Habitat and diet of wild naked mole-rats

According to continuous field observations, the main habitat of the naked mole-rat is characterized by arid vegetation such as shrubs and desert bush covered areas, which is located in arid zones of the Eastern and Southern Ethiopia. Naked mole-rat colonies dig extensive underground burrow systems, which they rarely leave. The type of diet consumed by the rodents was assessed. Naturally occurring plant species were found to be rich in flavonoids, essential fatty acid, solanine alkaloids, carotenoids, tannins, starch, fiber, vitamins, cafeic acid derivatives and others [20–32] (Additional file 1: Table S1).

### Innate immune cytokine profiles upon stimulation of human whole blood by microbes of the naked mole-rat

**Fig. 1:**
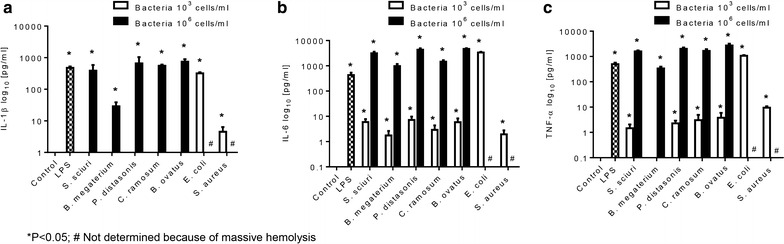
Stimulation of inflammatory cytokines by naked mole-rat microbes. Heparinized blood from healthy volunteers was stimulated by medium alone (negative control), LPS (positive control), *S.* *sciuri*, *B. megaterium*, *P. distasonis*, *C. ramosum*, *B.* *ovatus* and two human pathobionts *E. coli* and *S. aureus* at 37 °C and 5 % CO_2_ for 8 h. The levels of the cytokines IL-1β (**a**), TNF-α (**b**), IL-6 (**c**) were measured by cytometric bead array assays. The experiment was done in triplicates and data are expressed as mean ± SEM. *p < 0.05; ^#^strong hemolysis

Microbes are known to cross the gut barrier and possibly induce inflammatory responses in different organs [4]. Therefore, we asked to what extent microbes of the naked mole-rat are capable to stimulate the innate immune system of humans in an ex vivo setting in comparison to bacteria present in the human gut. For that heparinized human blood was mixed with a defined number of microbial cells, specifically *B. megaterium*, *S. sciuri*, *P. distasonis*, *C. ramosum* and *B. ovatus* at low (10^3^ cells/mL) and high (10^6^ cells/mL), concentrations respectively, and incubated at 37 °C for 8 h. Stimulation by 10 ng LPS was carried on to verify the viability and responsiveness of the white blood cells. Accordingly, at a low concentration of naked mole-rat bacterial cells no release of cytokines could be observed as exemplified for interleukin-1ß (IL-1ß) (Fig. 1). In contrast, using equivalent numbers of the pathobionts *E. coli* and *S. aureus*, an augmented release of IL-1β versus control was observed (p < 0.05). Likewise, a similar trend was seen with respect to tumor necrosis factor-α (TNF-α) and low concentrations of gut microbes. As expected, stimulation by *E. coli* and *S. aureus* induced a remarkable release of TNF-α from human white blood cells versus control (p < 0.05). In contrast, a low number of microbes was found to significantly induce IL-6, except *B. megaterium*. Again, this contrasts the strong effect of *E. coli* on this inflammatory cytokine (p < 0.05).

Higher number of microbes causes an enhanced release of inflammatory cytokines as manifested for IL-1β, TNF-α, and IL-6 (p < 0.05) respectively, irrespective of the type of isolates (Fig. 1a–c). With exception of *B. megaterium* (p < 0.05) all other isolates caused an elevated response of IL-1β and TNF-α. Moreover, *B. megaterium* was found to induce less IL-6 release compared to other microbes. Unfortunately, high concentration of *E. coli* and *S. aureus* could not be tested as they caused massive hemolysis of the blood that resulted in the incapability of the cells to secret cytokines.

## Discussion

The human intestine is believed to contain approximately 100 trillion intestinal (gut) microbiota comprising about 500–1000 different species [33]. These intestinal microbiota exist in a symbiotic relationship with their host, by metabolizing compounds that the host is unable to utilize and controlling the immune balance of the host’s body. However, the composition of the intestinal microbiota is known to vary, depending on diet, nutritional status, and other factors. It has been recognized that the intestinal microbiota is involved in the pathogenesis of diverse diseases not only in the intestine but also in organs distant from the intestine. Thus, the intestinal microbiota might contribute to the onset of diseases such as cancer by the pro-carcinogenic activities of specific pathogens and by synthesis of bacterial metabolites circulating in the host’s body, as well. For instance, mice supplemented orally with certain bacteria in early life were resistant to oncogene-associated mammary carcinogenesis later in life [34].

Resistance to cancer and extraordinary life span are hallmarks of the naked mole-rat [35]. Therefore, it was our aim to investigate the composition of the cultivable microbiota of these animals. Our results show that in wild naked mole-rats, Firmicutes and Bacteroidetes were consistently found in highest number. Similarly, previous studies in humans suggested that Firmicutes and Bacteroides are among the dominant enterotypes of the gut microbiota of most mammals across a wide range of species [36]. For the naked mole-rat we found a ratio of Firmicutes to Bacteroidetes of 8/1, which is similar to one found in healthy human adults of 10/1 [15]. For comparison, in obese adults the ratio rises to 100/1 and drops to 1/1 in people with chronic inflammatory bowel disease [37]. Interestingly, the phylum Proteobacteria, which comprises a wide range of potential pathogens [38] was among the less identified microbiota of the naked mole-rat. Moreover, most microbes identified were found to be the normal flora, suggesting that the animals harbor healthy-like microbiota.

The link between microbiota and aging is still poorly understood. However, it has been reported that age-related differences in the microbiota composition could be associated with immunosenescence [39] and increased frailty [40]. A recent study has shown increased microbial loads in aged *Drosophila*, which was associated with age-related dysplasia [41]. In contrast to human and mice that harbor 10^8^–10^12^ and 10^6^–10^9^ c.f.u./g, respectively [42, 43], the quantity of the microbiota of the naked mole-rat was lower, ranging from 10^2^ to 10^5^ c.f.u./g when verified by cultivation. However, diet intervention could potentially have effect in a general population of microorganism of the rodent [44]. We may also expect a considerable higher diversity of microbes when using more sensitive approaches such as 16S rRNA sequencing.

Early in life, exposure to microbes is known to shape the immune system [45]. The gastrointestinal tract immune network includes neutrophils and regulatory T-cells which communicate with the commensal microbiota. Recent data suggest that commensal bacteria–host crosstalk is continuous and reciprocal throughout life [46]. Enrichment of facultative anaerobes, notably pathobionts, is associated with an enhanced inflammatory status, as determined by inflammatory markers such as IL-6 and IL-8 in blood. Especially, chronic inflammation is known to be associated with metastasizing of tumor cells [47].

These finding forced us to analyze the microbes of the naked mole-rats’ intestine with respect to their capability of stimulating inflammatory cytokines in blood. Overall, naked mole-rat microbes were found to be less stimulatory of inflammatory cytokines like IL-1β, IL-6 and TNF-α compared with pathobionts from human gut (Fig. 1). Notably, *B. megaterium* which constitutes the most abundant species (45.2 %) in the naked mole-rat intestine seems to induce the least inflammatory response. Whether this has biological implications for the animal’s health needs further investigation. Harboring of abundant *B. megaterium* in the gut of naked mole-rats might not be surprising as they are very likely to acquire it from the soil due to their subterranean lifestyle. This bacterium is a common gram-positive, mainly aerobic, spore forming, soil bacterium, which is currently widely used in the field of biotechnology for recombinant protein production [48]. *B. megaterium* is used to produces penicillin amidase (essential for the synthesis of β-lactam antibiotics), various amylases, pyruvate, vitamin B12, as well as further unusual enzymes and components, which provide various health benefits such as playing a key role in several metabolic pathways, as endogenous scavenger of certain reactive oxygen species, being involved in DNA repair and synthesis, epigenetic gene regulation, and possessing antifungal and antiviral properties [49–51]. In addition, *Paenibacillus* spp. was among frequently identified isolates from the gut of naked mole-rats which are known to produce a polymyxin-like antibiotic that is effective against most gram-negative bacteria [52]. This might suggest that *B. megaterium* and *Paenibacillus* spp. are beneficial gut symbionts of naked mole-rat. Thus, the presence of these bacteria may probably contribute to the naked mole-rat’s resistance to various diseases, which however, needs to be proved yet.

In humans first colonization of the gut occurs during birth. In naked mole rat there is an additional delivery of bacteria to the pups by coprophagy [8]. This may provide pups with endosymbiotic gut flora and transitional source of food. Against this background and the life in a very confined space it is hard to understand the microbial diversity found in the animals in our study. However, as deduced from human studies it is quite possible that difference in gut microbial composition may have impact on shaping the behavior of individuals in the colony [53].

Our field study of natural food sources shows that this long-lived rodent has adapted to consume a wide variety of different plant species, many of which contain large quantities of polyphenols. Furthermore, the content and medical importance of each plant were systematically reviewed and shows that the plant-derived diet of wild naked mole-rats is rich in various anti-oxidants, anti-inflammatory, anti-cancer and anti-microbial agents (Additional file 1: Table S1). Despite the fact that the underlying mechanisms are not yet fully understood, recent evidence exists that polyphenols may actively contribute to the prevention of certain illnesses such as cardiovascular and chronic intestinal diseases. Likewise, many studies emphasize the role of polyphenols in prevention of oxidative stress in the pathogenesis of age-related human diseases. Polyphenols have been shown to scavenge free radicals and protect cell constituents against oxidative damage [54]. Oral administration of polyphenols to rats limits DNA oxidative damage in caeca mucosal cells and can act as a pro-oxidant, thereby inducing apoptosis and reducing the tumor incidence and growth [55–57]. However, the relationship between phenolic compounds and microbiota in terms of health benefits is still poorly understood [58]. Nonetheless, polyphenols have been shown to inhibit the adherence of potentially pathogenic microbes to host cells, while enhancing the proliferation and adhesion of beneficial probiotic bacteria thereby contributing to sustain gut health [59]. Additionally, the consumption of polyphenols has a prebiotic effect on the gut microbiota [60]. In this line, the polyphenol-rich natural diet of wild naked mole-rats in combination with various other plant constituents might contribute to their astonishing resistance to various diseases and their healthy ageing. Therefore, it is not surprising, that as far as longevity is concerned, recent studies revealed that the microbiota composition in individual ‘centenarianas’ showed tenfold increase in *Eubacteriumlimosum* [39, 61] that is an anaerobic acetogenic bacterium producing acetate, butyrate, ethanol and vitamin B12.

## Conclusion

In summary, the cultivable gut flora of the naked mole-rat is composed of a microbiota that is low in number, but diverse and dominated mainly by *B. megaterium.* Our finding that naked mole-rats consume polyphenol-rich plants suggests that it could exert a protective effect against different diseases and aging.

## Additional file

10.1186/s13099-016-0107-3 Biochemical contents of food-plants consumed by naked mole-rats in the wild and their medical significance. The table demonstrates naturally occurring plant species consumed by the rodent in the wildlife and bioactive constituents of the plants including their medical significance. Naked mole-rats were obtained from wild and housed in a colony of 20 animals. Feeding trials were done by offering of different fresh plant species that are proximate to the habitat of the animal. The biochemical constituents and their medicinal importance of naturally occurring plant species consumed by the rodent in the wild were systematically reviewed. The main diet of the animal is assigned with double asterisk (**) and rarely consumed plant species are those with a single asterisk (*).

## Abbreviations

LPS: lipopolysaccharide
RPMI: Roswell Park Memorial Institute
CBA: cytometry bead array
c.f.u.: colony-forming unit
spp.: species
FACS: fluorescence-activated cell sorting
MALDI-TOF: matrix-assisted laser desorption ionization time-of-flight
CHCA: α-cyano-4-hydroxycinnamic acid
IL: interleukin
TNF: tumor necrosis factor
