# Supplementary material for: Analysis of cultivable microbiota and diet intake pattern of the long-lived naked mole-rat
Source: Gut Pathog. 2016 May 28;8:25. doi: 10.1186/s13099-016-0107-3 (PMC4884373; doi:10.1186/s13099-016-0107-3)
Supplement: Supplementary file 1 — 10.1186/s13099-016-0107-3 Biochemical contents of food-plants consumed by naked mole-rats in the wild and their medical significance. The table demonstrates naturally occurring plant species consumed by the rodent in the wildlife and bioactive constituents of the plants including their medical significance. Naked mole-rats were obtained from wild and housed in a colony of 20 animals. Feeding trials were done by offering of different fresh plant species that are proximate to the habitat of the animal. The biochemical constituents and their medicinal importance of naturally occurring plant species consumed by the rodent in the wild were systematically reviewed. The main diet of the animal is assigned with double asterisk (**) and rarely consumed plant species are those with a single asterisk (*). [file 13099_2016_107_MOESM1_ESM.docx]

Table S1 Biochemical contents of food-plants consumed by naked mole-rats in the wild and their medical significance

| Local name | Species name | Family | Constituents | Medicinal value |
| --- | --- | --- | --- | --- |
| Hargessaa | *Aloe trichosantha** | Xanthorrhoeaceae | flavonoids, anthranoids, lectins, galactomannans | free radical scavengers, anti-inflammatory, anti-cancerogenic, anti-microbial |
| Hiddii | *Solanum incanum** | Solanaceae | flavonoid solanin and other solanum alkaloids, caffeic acid ester | antimicrobial, anti-inflammatory, anti-cancerogenic, free radical scavengers |
| Coppii | *Cissus quadrangularis*** | Vitaceae | carotenoids, flavonoids, essential fatty acids , vitamin E, phytosterols | antioxidant, anti-inflammatory, anti-cancerogenic, anti-microbial, decreasing blood sugar |
| Saarreettii | *Asparagus africanus** | Asparagaceae | tannin, steroidsaponins, lignans | free radical scavengers, antiprotozoal |
| Ameesaa | *Commiphora Africana*** | Burseraceae | sesquiterpenes, flavonoids, polysaccharides | free radical scavengers, anti-inflammatory, anti-cancerogenic, anti-microbial |
| Burkukkee | *Acacia nilotica*** | Fabaceae | triterpenoid saponins, tannins, flavonoids | stabilizing blood vessels, free radical scavengers, anti-inflammatory, anti-cancerogenic, anti-microbial |
| Urgoo gabaaboo | *Endostemon tenuiflorus*** | Lamiaceae | cafeic acid derivates | free radicals scavengers |
| Hamareesaa | *Acacia brevispica** | Fabaceae | proanthocyanidins, catechol tannins, gallíc acid, chlorogenic acid, flavonoids | free radical scavengers, anti-inflammatory, anti-cancerogenic, anti-microbial |
| Maxaaxisha | *Ipomoea batatas*** | Convolvulaceae | starch, fibers, vitamins, carotenoids | antioxidant, prebiotic |
| Lewizii | *Arachis hypogaea*** | Fabaceae | fiber, phytic acid , trace elements , arginine, unsaturated fatty acids | DNA repair, prebiotic |

Sources [20-32].

The table demonstrates naturally occurring plant species consumed by the rodent in the wildlife and bioactive constituents of the plants including their medical significance. Naked mole-rats were obtained from wild and housed in a colony of 20 animals. Feeding trials were done by offering of different fresh plant species that are proximate to the habitat of the animal. The biochemical constituents and their medicinal importance of naturally occurring plant species consumed by the rodent in the wild were systematically reviewed. The main diet of the animal is assigned with double asterisk (**) and rarely consumed plant species are those with a single asterisk (*).
